# Supplementary material for: Active DNA damage response signaling initiates and maintains meiotic sex chromosome inactivation
Source: Nat Commun. 2022 Nov 28;13:7212. doi: 10.1038/s41467-022-34295-5 (PMC9705562; doi:10.1038/s41467-022-34295-5)
Supplement: Supplementary file 1 — Supplementary Information [file 41467_2022_34295_MOESM1_ESM.pdf]

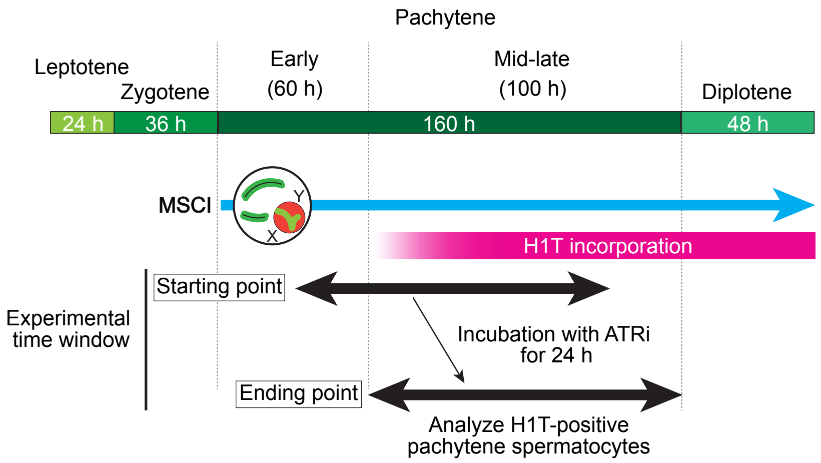

Supplementary Fig. 1: Meiotic progression and experimental design of this study.
In mice, the duration of the pachytene stage is approximately 160 h, and the mid-late pachytene stage, where H1T incorporation takes place, lasts approximately 100 h[32]. Because MSCI is established at the onset of the pachytene stage and the duration of the early pachytene stage is approximately 60 h, H1T-positive spermatocytes after 24 h culture, have already initiated MSCI at the time the culture was started.

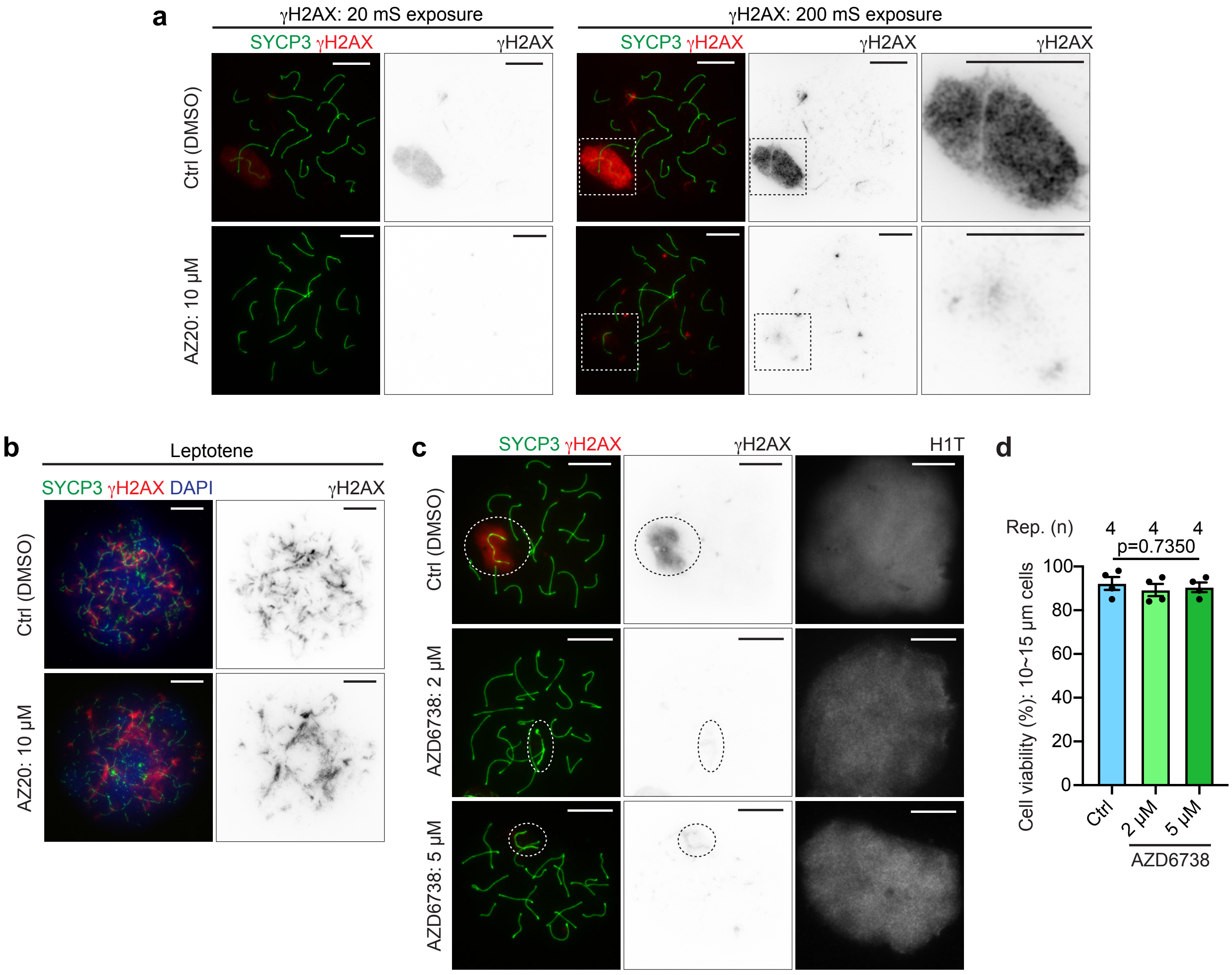

Supplementary Fig. 2: Evaluation of the effects of AZ20 and an alternative ATR inhibitor AZD6738 on the γH2AX domain.

Chromosome spreads of mid-late pachytene spermatocytes immunostained with antibodies raised against SYCP3 (**a, b, c**), γH2AX (**a, b, c**), and H1T (**c**). Representative images of 3 independent experiments are shown.

a. γH2AX signal was captured utilizing a shorter exposure (20 mS) and a longer exposure (200 mS) to confirm attenuated γH2AX. XY chromosomes are indicated with dashed squares and are magnified in the panels to the right. With a 20 mS exposure, although γH2AX signals were visible in the control, γH2AX signals were not visible in spermatocytes treated with AZ20. A 200 mS exposure detected weak γH2AX signals on the XY chromatin in spermatocytes treated with AZ20, while the control showed a strong γH2AX domain on the XY.

b. Exposure to 10 µM AZ20 for 24 h did not affect γH2AX formation in spermatocytes at the leptotene stage where ATM is the primary kinase responsible for generating γH2AX, thereby confirming the specificity of AZ20 for inhibiting signaling by the ATR-dependent DDR. XY chromosomes are indicated with dashed circles.

c, d. Incubation with AZD6738 at 2 or 5 µM also significantly attenuated the maintenance of γH2AX on the XY in mid-pachytene, but did not affect the viability of spermatocytes after a 24 h incubation. One-way ANOVA for 4 independent experiments (**d**).

Scale bars: 10 µm.

Source data are provided as a Source Data file.

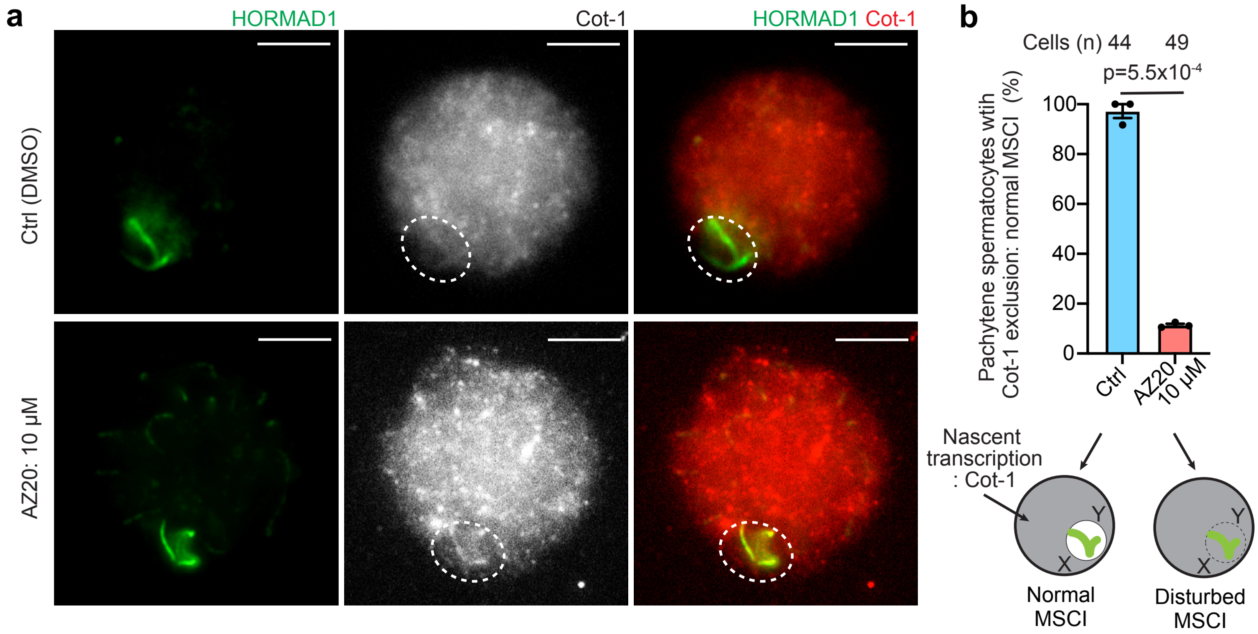

Supplementary Fig. 3: Evaluation of MSCI by Cot-1 RNA FISH.

a. Cot-1 RNA FISH combined with immunostaining with antibodies raised against HORMAD1, which detects XY axes. XY chromosomes are indicated with dashed lines. Single Z-sections are shown. Scale bars: 10 μm.

b. Quantification of mid-late pachytene spermatocytes with a normal Cot-1 RNA FISH signals on the XY chromosomes shown as the mean ± s.e.m. for 3 independent experiments. Total numbers of analyzed nuclei are indicated in the panel. Two-tailed unpaired t-test.

Source data are provided as a Source Data file.

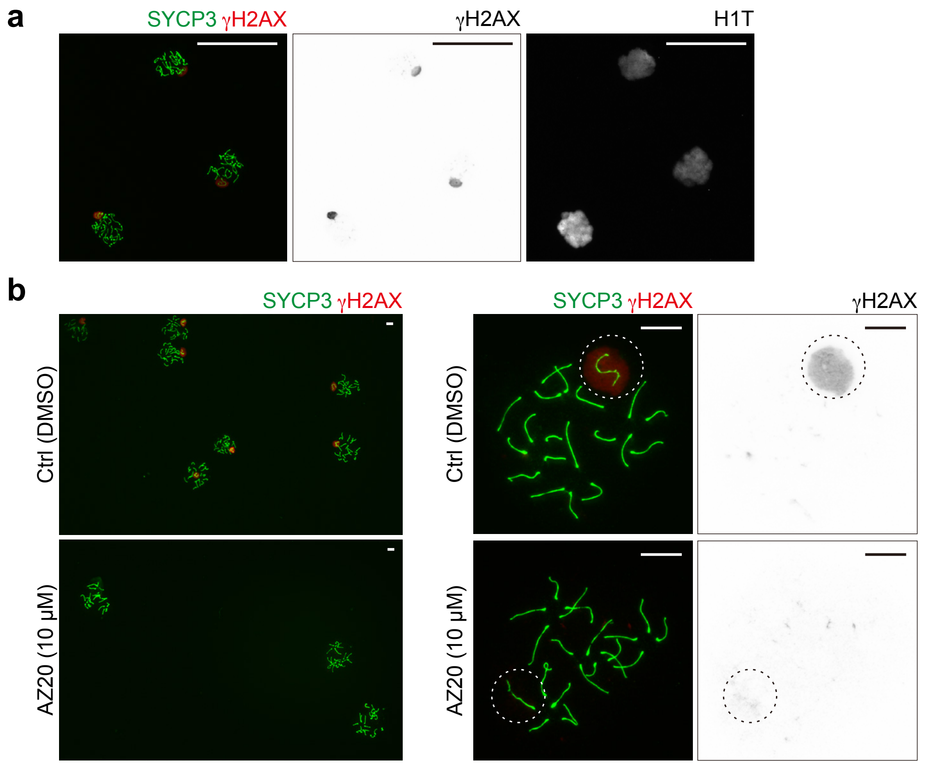

c

	Isolated H1T-positive PS by FACS	Treatment	Viability	γH2AX domain on XY	Cell No. applied for RNA-seq
Rep. 1	> 90 %	Ctrl	100%	> 90%	15,000 cells
		AZ20 10 µM	100%	10% >	15,000 cells
Rep. 2	> 90 %	Ctrl	96%	> 90%	15,000 cells
		AZ20 10 µM	97%	10% >	15,000 cells

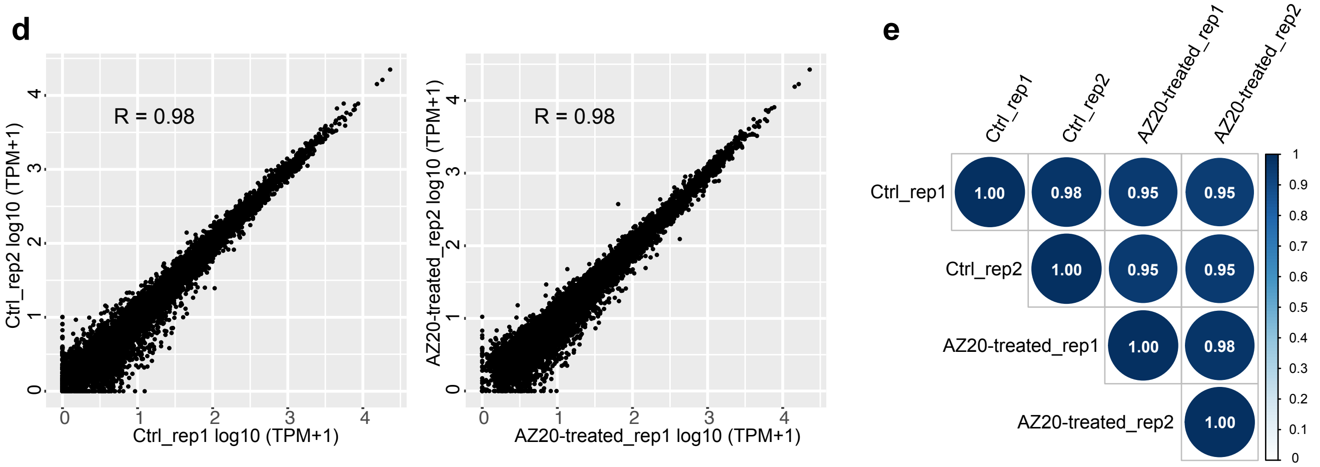

Supplementary Fig. 4: The purity of isolated pachytene spermatocytes utilized for RNA-seq analyses.
a. Chromosome spreads of spermatocytes isolated using FACS. Samples were immunostained with antibodies raised against SYCP3, γH2AX and H1T. Representative images of two independent experiments are shown.
b. Chromosome spreads of pachytene spermatocytes cultured with or without AZ20 for 24 h were immunostained with antibodies raised against SYCP3 and γH2AX. Representative images taken by higher magnification are shown in the panels to the right. XY chromosomes are indicated with dashed circles. Representative images of two independent experiments are shown.
Scale bars: 10 µm.
c. Summary of the purities of spermatocytes utilized for RNA-seq in Fig. 2.
d. Scatter plots showing the reproducibility of gene expression between biological replicates in each experimental group. R values indicate the Pearson correlation coefficient. The left panel is a plot for the Ctrl and the right panel is a plot for AZ20-treated spermatocytes.
e. Heatmap showing Pearson correlation coefficient values between each sample.
Source data are provided as a Source Data file.

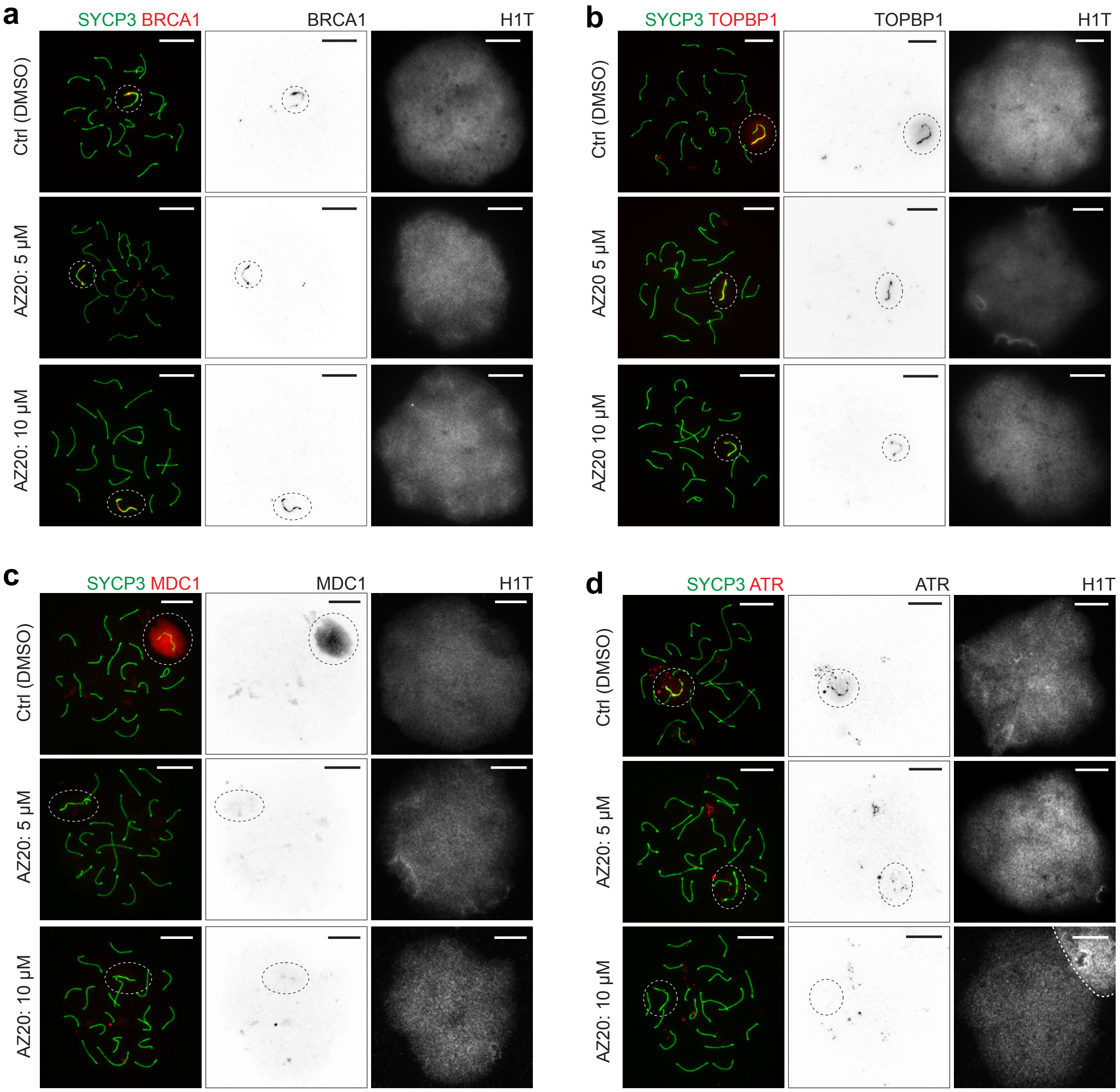

Supplementary Fig. 5: Additional examples of the effects of ATRi on the localization of various DDR factors on XY chromosomes in mid-late pachytene spermatocytes.

Chromosome spreads of mid-late pachytene spermatocytes immunostained with antibodies raised against BRCA1 (**a**), TOPBP1 (**b**), MDC1 (**c**), or ATR (**d**), all of which are also immunostained using antibodies against SYCP3 and H1T to identify the XY axes and mid-late pachytene spermatocytes, respectively. Representative images of 3 independent experiments are shown. XY chromosomes are indicated with dashed circles (**a-d**). The boundary between two different spermatocytes is shown with a dashed line (**d**). Exposure to 5 µM AZ20 for 24 h showed similar changes in the localization of DDR factors as exposure to 10 µM AZ20 for 24 h (shown in Figure 3), as explained in the main text.

Scale bars: 10 µm.

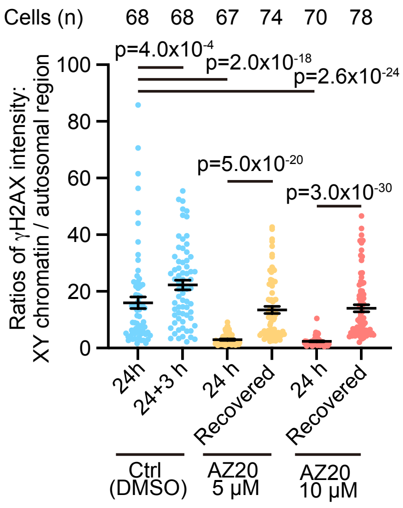

Supplementary Fig. 6: Reestablishment of DDR domain with 3 h recovery after release from ATR-inhibiton by AZ20.
Ratios of γH2AX intensity on XY chromatin compared with it on autosomal region shown as the mean ± s.e.m. for 3 independent experiments.
Two-tailed Mann Whitney test.

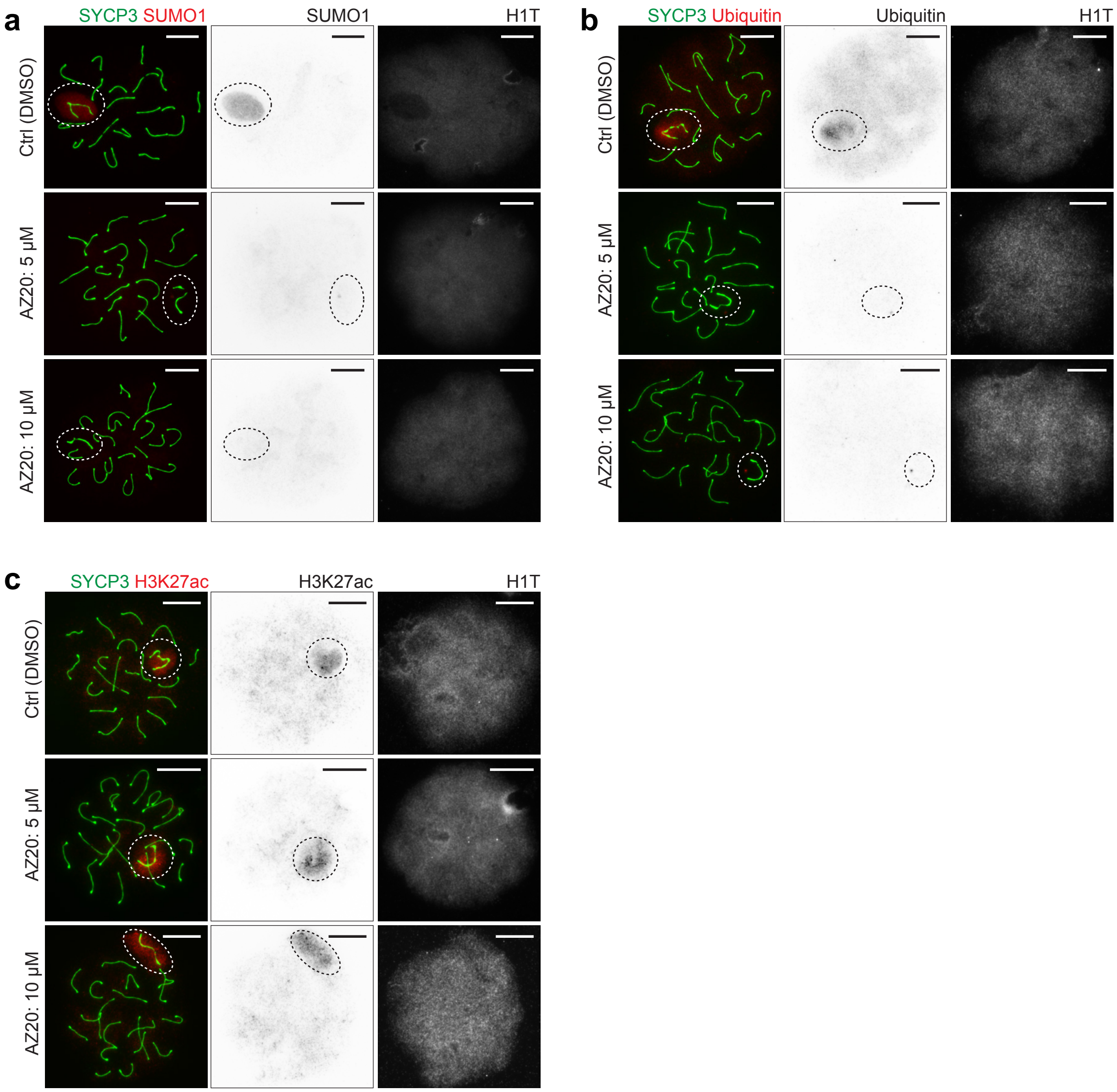

Supplementary Fig. 7: Images for various post-translational modifications established on the XY chromatin. Chromosome spreads of mid-late pachytene spermatocytes immunostained with antibodies raised against SYCP3, H1T and SUMO1 (**a**), ubiquitin (**b**), or H3K27ac (**c**). Representative images of 3 independent experiments are shown. XY chromosomes are indicated with dashed circles. Exposure to 5 µM AZ20 and 10 µM AZ20 for 24 h showed the same changes in the localization of specific PTMs, as explained in the main text. SYCP3 and H1T indicate XY chromosome axes and mid-late pachytene spermatocytes, respectively.
Scale bars: 10 µm.

a

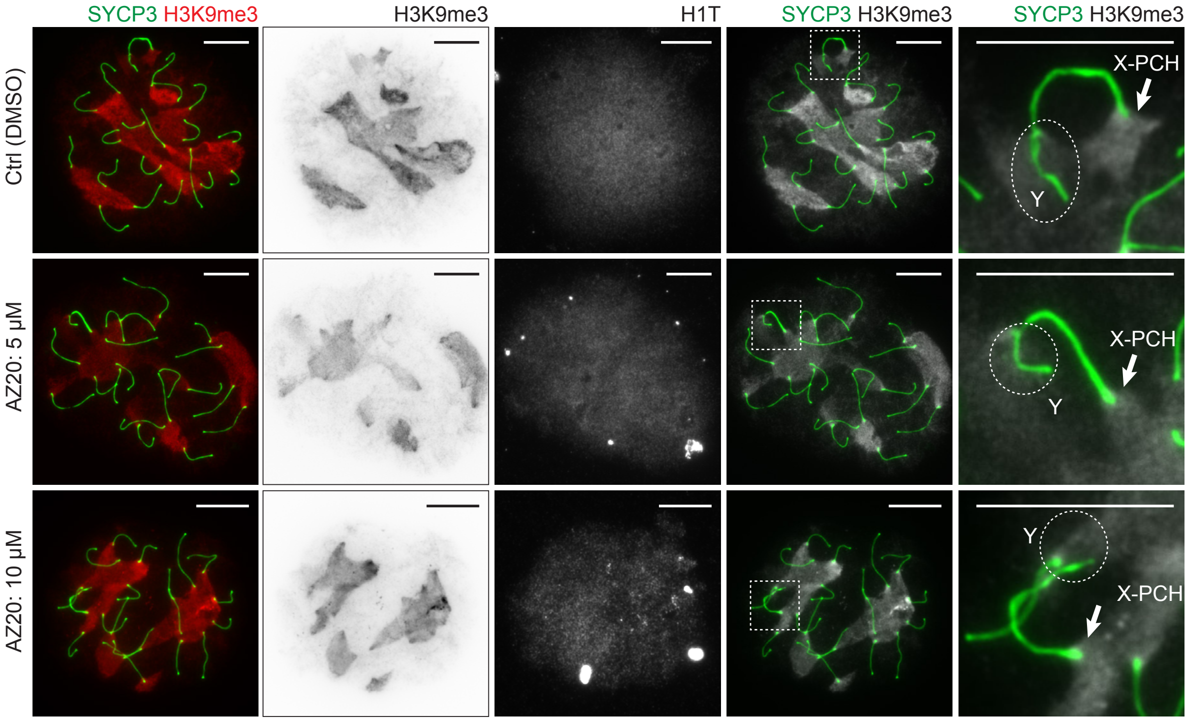

b

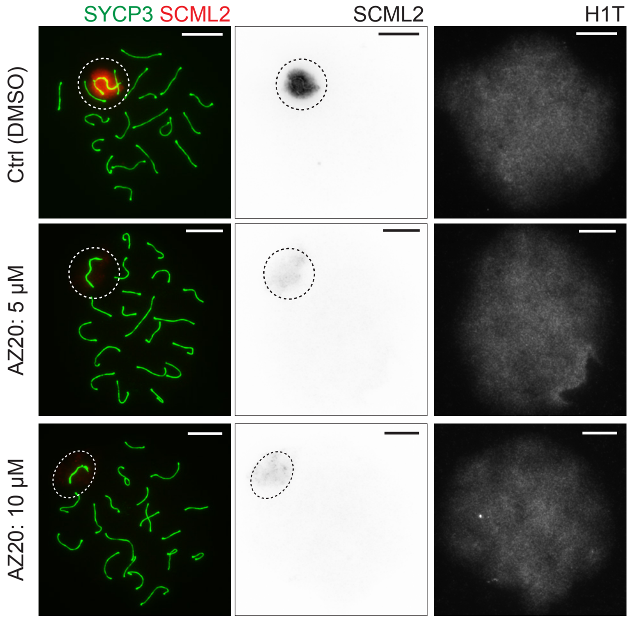

Supplementary Fig. 8: Localization of H3K9me3 and SCML2 on the XY chromatin.
a. Chromosome spreads of mid-late pachytene spermatocytes immunostained with antibodies raised against SYCP3, H3K9me3, and H1T. Representative images of 3 independent experiments are shown. XY chromosomes are indicated with dashed squares and are magnified in the panels to the right. ATRi treatment did not affect the localization of H3K9me3 to X-PCH and the Y chromosome.
b. Chromosome spreads of mid-late pachytene spermatocytes immunostained with antibodies raised against SYCP3, SCML2, and H1T. Representative images of three independent experiments are shown. XY chromosomes are indicated with dashed circles. ATRi treatment altered SCML2 localization, suggesting that retention of SCML2 localization is dependent on active DDR signaling.
X-PCH: X-pericentromeric heterochromatin. Scale bars: 10 µm.

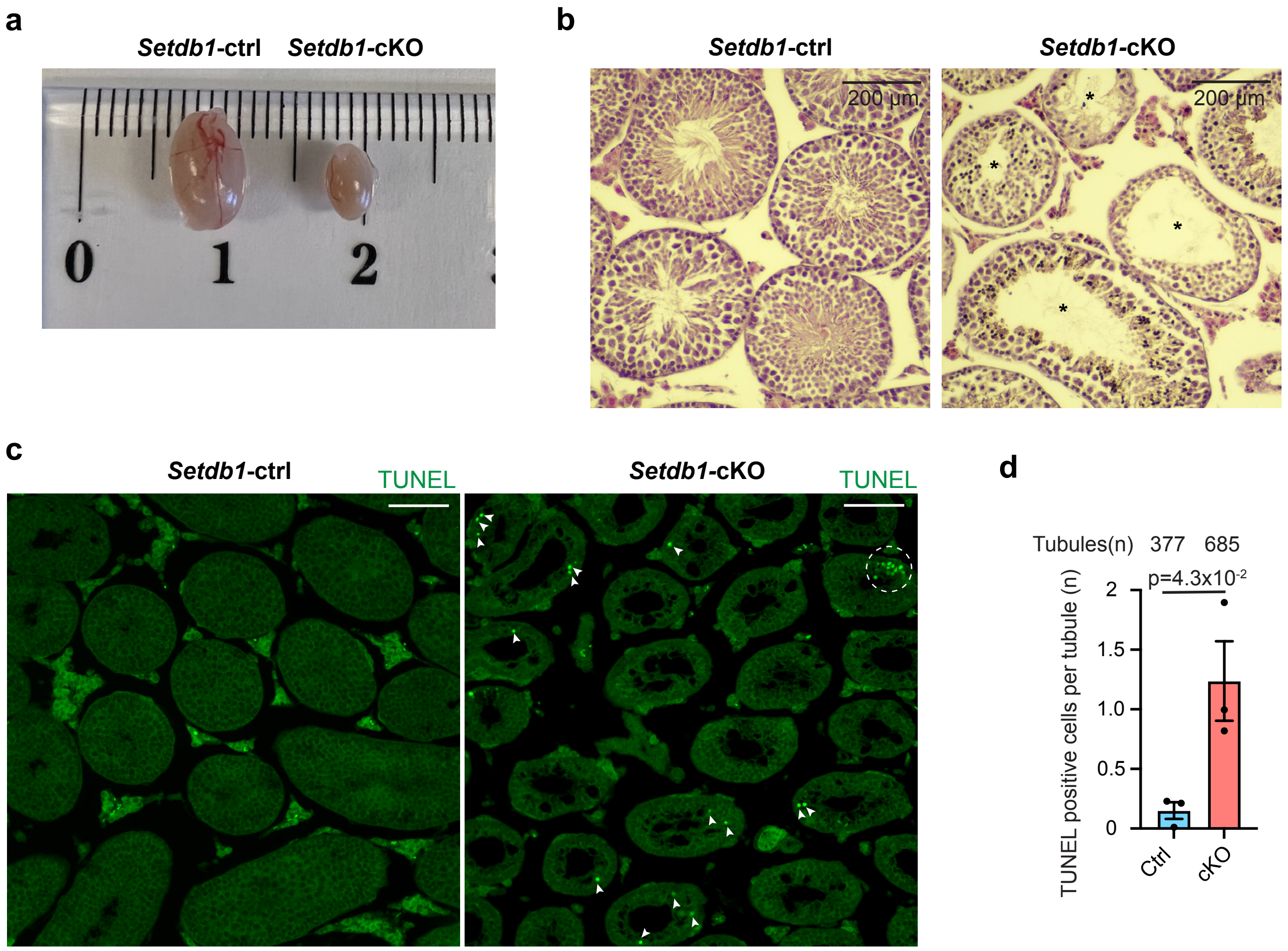

Supplementary Fig. 9: Phenotypes of *Setdb1*-cKO testes.

a. A photo of testes from *Setdb1*-ctrl and *Setdb1*-cKO males at 4 months-of-age.

b. Histology of testis sections from *Setdb1*-ctrl and *Setdb1*-cKO males at 4 months-of-age, stained with hematoxylin & eosin. Three independent mice of each genotype were used for analysis and representative images are shown. Star signs indicate the germ cell loss in the tubules.

c. Testis sections from *Setdb1*-ctrl and *Setdb1*-cKO males at 3 weeks-of-age; apoptotic events were detected by TUNEL assay. White arrowheads indicate apoptotic nuclei, and the dashed circle indicates a region containing a cluster of apoptotic nuclei.

d. Quantification of TUNEL positive cells per tubule shown as the mean ± s.e.m. for 3 independent experiments. The total numbers of tubules analyzed are indicated in the panels. Two-tailed unpaired t-tests.

Scale bars: 200 μm.

Source data are provided as a Source Data file.

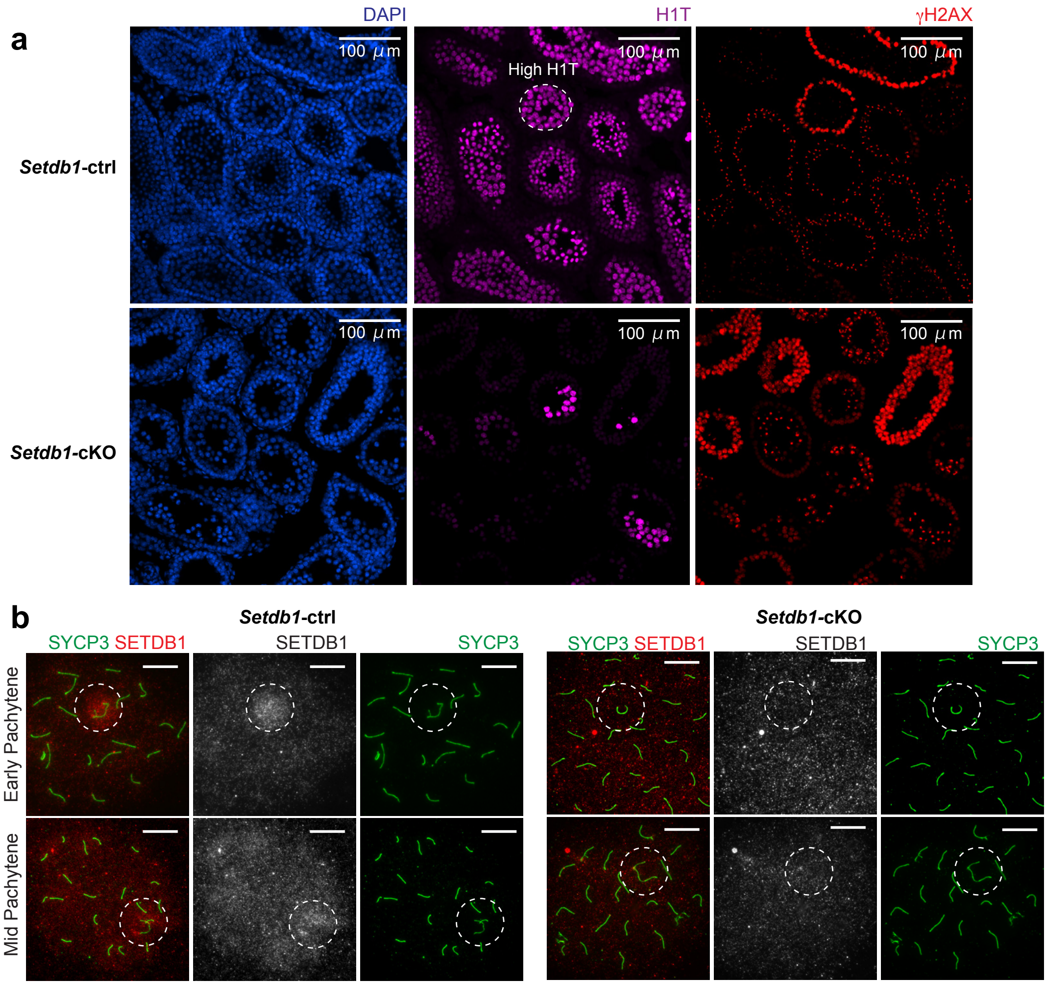

Supplementary Fig. 10: Validation of meiotic arrest and depletion of SETDB1 in *Setdb1*-cKO spermatocytes.

a. Testis sections from 3-weeks old mice immunostained with antibodies raised against γH2AX and H1T. Representative images of 3 independent experiments are shown. Meiotic arrest in the *Setdb1*-cKO was confirmed by the lack of late spermatogenetic cells; in contrast, controls strongly express H1T as an indicator of meiotic progression. A seminiferous tube that contains late pachytene spermatogenic cells (high H1T) is indicated with a dashed circle.

b. Chromosome spreads of early and mid pachytene spermatocytes immunostained with antibodies raised against SYCP3 and SETDB1. Representative images of 3 independent experiments are shown. XY chromosomes are indicated with dashed circles. These images confirm the depletion of SETDB1 protein from XY chromatin at early and mid pachytene in *Setdb1*-cKO spermatocytes.

Scale bars: 10 μm unless otherwise described in the panels.

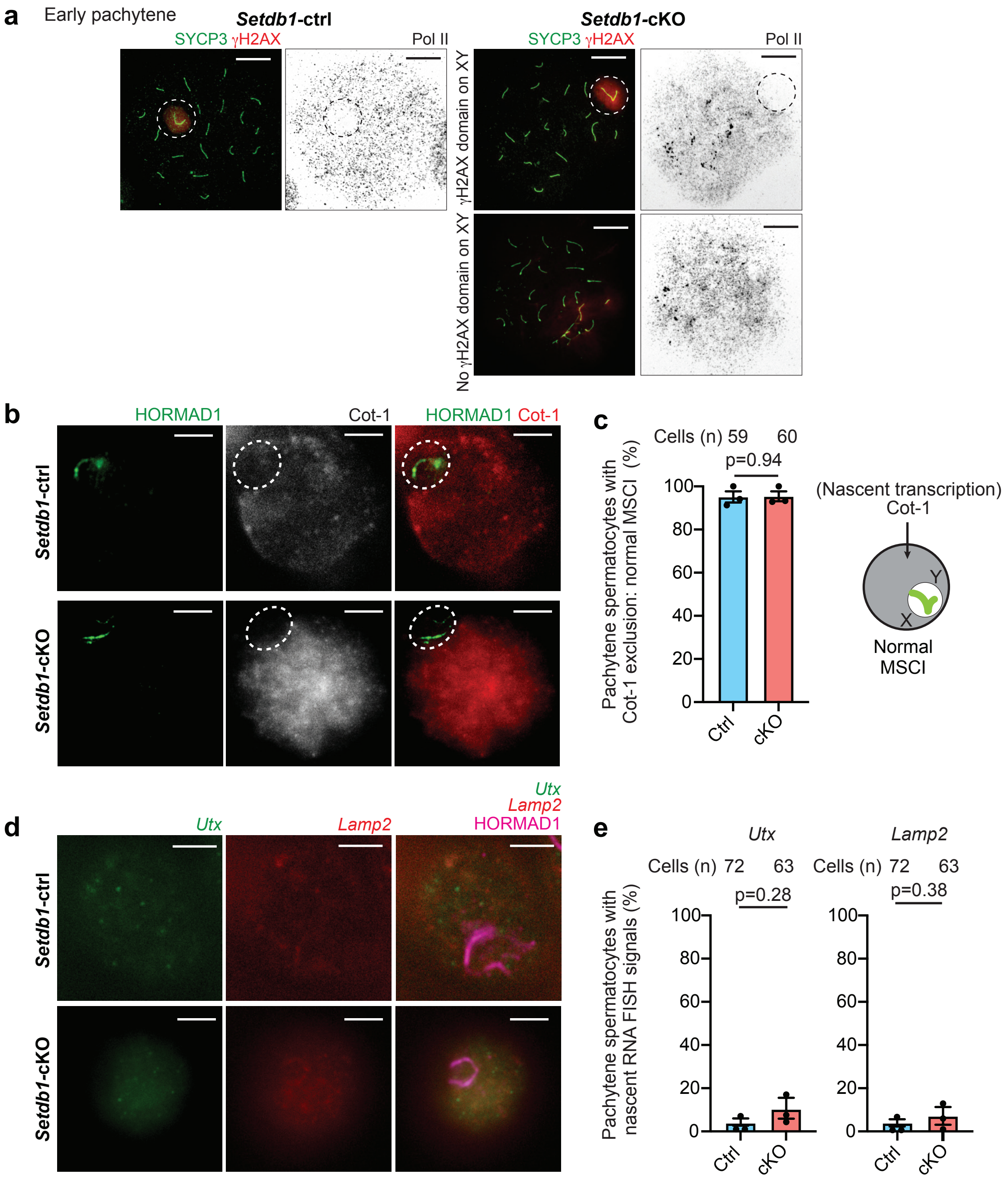

Supplementary Fig. 11: Evaluation of MSCI in *Setdb1*-cKO spermatocytes.

a. Chromosome spreads of early pachytene spermatocytes immunostained with antibodies raised against SYCP3, γH2AX, and Pol II. XY chromosomes are indicated with dashed circles. In *Setdb1*-cKO spermatocytes, the presence of a normal γH2AX domain on XY chromatin and exclusion of Pol II from XY chromatin was confirmed in early pachytene spermatocytes, as we show for mid pachytene spermatocytes in the main figures; it should be noted that a portion of pachytene spermatocytes (~20 %) showed defective synapsis formation and thereby lack the γH2AX domain.

b. Cot-1 RNA FISH on mid-late pachytene spermatocytes using 3D slides. XY axes were detected by immunostaining with antibodies raised against HORMAD1. XY chromosomes are indicated with dashed lines. Single Z-sections are shown.

c. Quantification of mid-late pachytene spermatocytes with a normal Cot-1 RNA FISH on the XY shown as the mean ± s.e.m. for 3 independent experiments.

d. Gene-specific RNA FISH for the X-linked *Utx* and *Lamp2* genes. XY axes were detected by immunostaining with antibodies raised against HORMAD1. Single Z-sections are shown.

e. Quantification of mid-late pachytene spermatocytes with nascnet RNA FISH signals for *Utx* (left panel) and *Lamp2* genes (right panel) shown as the mean ± s.e.m. for 3 independent experiments.

Total numbers of nuclei analyzed are indicated in the panels (**c**,**e**). Two-tailed unpaired t-tests (**c**,**e**). Scale bars: 10 µm.
