# Supplementary material for: Active DNA damage response signaling initiates and maintains meiotic sex chromosome inactivation
Source: Nat Commun. 2022 Nov 28;13:7212. doi: 10.1038/s41467-022-34295-5 (PMC9705562; doi:10.1038/s41467-022-34295-5)
Supplement: Supplementary file 3 — Reporting Summary [file 41467_2022_34295_MOESM3_ESM.pdf]

nature portfolio

Corresponding author(s): Satoshi H Namekawa, Hironori Abe

Last updated by author(s): Oct 8, 2022

# Reporting Summary

Nature Portfolio wishes to improve the reproducibility of the work that we publish. This form provides structure for consistency and transparency in reporting. For further information on Nature Portfolio policies, see our Editorial Policies and the Editorial Policy Checklist.

## Statistics

For all statistical analyses, confirm that the following items are present in the figure legend, table legend, main text, or Methods section.

| n/a | Confirmed | |
|---|---|---|
| ☐ | ☒ | The exact sample size (*n*) for each experimental group/condition, given as a discrete number and unit of measurement |
| ☐ | ☒ | A statement on whether measurements were taken from distinct samples or whether the same sample was measured repeatedly |
| ☐ | ☒ | The statistical test(s) used AND whether they are one- or two-sided<br>*Only common tests should be described solely by name; describe more complex techniques in the Methods section.* |
| ☒ | ☐ | A description of all covariates tested |
| ☒ | ☐ | A description of any assumptions or corrections, such as tests of normality and adjustment for multiple comparisons |
| ☐ | ☒ | A full description of the statistical parameters including central tendency (e.g. means) or other basic estimates (e.g. regression coefficient) AND variation (e.g. standard deviation) or associated estimates of uncertainty (e.g. confidence intervals) |
| ☐ | ☒ | For null hypothesis testing, the test statistic (e.g. *F*, *t*, *r*) with confidence intervals, effect sizes, degrees of freedom and *P* value noted<br>*Give P values as exact values whenever suitable.* |
| ☒ | ☐ | For Bayesian analysis, information on the choice of priors and Markov chain Monte Carlo settings |
| ☒ | ☐ | For hierarchical and complex designs, identification of the appropriate level for tests and full reporting of outcomes |
| ☐ | ☒ | Estimates of effect sizes (e.g. Cohen's *d*, Pearson's *r*), indicating how they were calculated |

*Our web collection on statistics for biologists contains articles on many of the points above.*

## Software and code

Policy information about availability of computer code

| | |
|---|---|
| Data collection | Image data were acquired  using ECLIPSE Ti-2 microscope (Nikon) and LSM800 confocal microscope (Zeiss). Intensity data were collected using Fiji. Sequencing data were collected using Illumina HiSeq X Ten. |
| Data analysis | Statistical data were analyzed by Prism 9. RNA-seq data were processed through trimmomatic (v0.39), STAR(version 2.5.4b), featureCounts (v2.0.l), DESeq2(version 1.36.0), corrplot (version. 0.92), ggplot2 (3.3.6). No custom code was used in this study. |

For manuscripts utilizing custom algorithms or software that are central to the research but not yet described in published literature, software must be made available to editors and reviewers. We strongly encourage code deposition in a community repository (e.g. GitHub). See the Nature Portfolio guidelines for submitting code & software for further information.

## Data

Policy information about availability of data

All manuscripts must include a data availability statement. This statement should provide the following information, where applicable:

- Accession codes, unique identifiers, or web links for publicly available datasets
- A description of any restrictions on data availability
- For clinical datasets or third party data, please ensure that the statement adheres to our policy

RNA-seq data reported in this study were deposited to the Gene Expression Omnibus (accession no. GSE211519). Source data are provided with this paper.

# Field-specific reporting

Please select the one below that is the best fit for your research. If you are not sure, read the appropriate sections before making your selection.

☒ Life sciences ☐ Behavioural & social sciences ☐ Ecological, evolutionary & environmental sciences

For a reference copy of the document with all sections, see nature.com/documents/nr-reporting-summary-flat.pdf

# Life sciences study design

All studies must disclose on these points even when the disclosure is negative.

| | |
|---|---|
| Sample size | Sample size was chosen to ensure reproducibility of the results at affordable costs while ensuring significance in statistical tests. Sample sizes are indicated for all experiments. At least 2 and typically 3 or more independent experiments were carried out for most of the assays. We examined three independent biological replicates for most of the analyses because three independent biological replicates were sufficient to confirm statistical significance. Bulk RNA-seq data were obtained from two independent biological replicates. |
| Data exclusions | No data were excluded from analyses. |
| Replication | We confirmed consistent results between three independent biological replicates for all experiments. Bulk RNA-seq data were obtained from two independent biological replicates. The consistency between replicates were examined by Pearson correlation and all datasets were included for analyses. |
| Randomization | Mature male C57BL/6 mice greater than 6-weeks old were randomly utilized for culture experiments. However, in analysis using mutant mice (Setdb1-cKO mice), randomization was not feasible since genotypes of the mice needed be determined. |
| Blinding | Blinding was not considered as the authors needed to be aware of conditions for the chemical treatment in culture and genotypes of the mice to perform all analysis. No blinding should not affect interpretation as all experiment measures were objective. |

# Reporting for specific materials, systems and methods

We require information from authors about some types of materials, experimental systems and methods used in many studies. Here, indicate whether each material, system or method listed is relevant to your study. If you are not sure if a list item applies to your research, read the appropriate section before selecting a response.

## Materials & experimental systems

| n/a | Involved in the study | |
|---|---|---|
| ☐ | ☒ | Antibodies |
| ☒ | ☐ | Eukaryotic cell lines |
| ☒ | ☐ | Palaeontology and archaeology |
| ☐ | ☒ | Animals and other organisms |
| ☒ | ☐ | Human research participants |
| ☒ | ☐ | Clinical data |
| ☒ | ☐ | Dual use research of concern |

## Methods

| n/a | Involved in the study | |
|---|---|---|
| ☒ | ☐ | ChIP-seq |
| ☒ | ☐ | Flow cytometry |
| ☒ | ☐ | MRI-based neuroimaging |

## Antibodies

| | |
|---|---|
| Antibodies used | This study made use of the following [format: host anti-protein (source or company with product/catalog number if applicable), dilution];<br>Primary antibodies<br>Rabbit anti-SYCP3 (Novus, NB300–232), 1/500; mouse anti-SYCP3 (Abcam, ab97672), 1/5000; goat anti-SYCP3 (R&D Systems, AF3750), 1/200; mouse anti-H2AX-pS139 (γH2AX: Millipore, 05–636), 1/5000; mouse anti-H2AX-pS139 (γH2AX) conjugated to Alexa 647 fluorophore (Millipore, 05–636-AF647), 1/2000; rabbit anti-TOPBP1 (a gift from Dr. Junjie Chen50), 1/2000; rabbit anti-BRCA1 (generated in the Namekawa Lab9), 1/500; sheep anti-MDC1 (Bio-Rad, AHP799), 1/500; guinea pig anti-H1T (gift from Dr. Mary Ann Handel), 1/2000; rabbit anti-ATR (Millipore, PC538), 1/2000; mouse anti-Pol II (Santa Cruz, sc-56767), 1/100; rabbit anti-H3K27ac (Active Motif, 39133), 1/2000; mouse anti-SUMO1 (Invitrogen, 33-2400), 1/200; rabbit anti-Ubiquitin (Abcam, ab19247), 1/200; mouse-H3K9me3 (Abcam, ab8898), 1/200; rabbit anti-SCML2 (generated in the Namekawa Lab32), 1/500; rabbit anti-SETDB1 (Proteintech, 11231-1-AP), 1/200; rabbit anti-HORMAD1 (gift from Dr. Attila Toth), 1/200.<br><br>Secondary antibodies (dilution: 1/500 in staining of chromosome spreads; 1/1000 in section staining)<br>Donkey anti-mouse IgG (H+L) Alexa Fluor 488 (Invitrogen, A-21202); donkey anti-rabbit IgG (H+L) Alexa Fluor 488 (Invitrogen, A21206); donkey anti-goat IgG (H+L) Alexa Fluor 488 (Invitrogen, A-11055); donkey anti-mouse IgG (H+L) Alexa Fluor 555 (Invitrogen, A-31570); donkey anti-rabbit IgG (H+L) Alexa Fluor 555 (Invitrogen, A-31572); donkey anti-sheep IgG (H+L) Alexa Fluor 555 (Invitrogen, A-21436); goat anti-guinea pig IgG (H+L) Alexa Fluor 555 (Invitrogen, A-21435); goat anti-guinea pig IgG (H+L) Alexa 647, (Jackson ImmunoResearch, 106-606-003). |

| | |
|---|---|
| Validation | All antibodies used in this study validated by the manufactures or by the Namekawa laboratory.<br>The specificity of the goat-anti SYCP3 antibody for IF was validated by manufacture (https://www.rndsystems.com/products/human-scp3-sycp3-antibody_af3750). The specificity of the mouse anti-H3K9me3 was validated by manufacture (https://www.abcam.com/histone-h3-tri-methyl-k9-antibody-chip-grade-ab8898.html).The specificities of the rabbit anti-H3K27ac and the rabbit anti-SCML2 were validated by the Namekawa lab (https://journals.plos.org/plosgenetics/article?id=10.1371/journal.pgen.1007233). The specificities of the rabbit anti-SETDB1 was validated by manufacture (https://www.ptglab.com/products/SETDB1-Antibody-11231-1-AP.htm). The specificities of the mouse anti-SUMO1 and the rabbit anti-Ubiquitin were validated in a previous study (https://www.science.org/doi/10.1126/science.aaf6407). The specificities of other antibodies used in this study were validated by the Namekawa lab (https://doi.org/10.1016/j.cub.2019.11.064). |

# Animals and other organisms

Policy information about studies involving animals; ARRIVE guidelines recommended for reporting animal research

| | |
|---|---|
| Laboratory animals | Mice were maintained on a 12h:12h light:dark cycle in a temperature and humidity-controlled vivarium (22 ± 2°C; 40–50% humidity) with free access to food and water in the pathogen-free animal care facility.<br>For culture experiments and AZ20 gavage treatments, mature male C57BL/6 (hereafter described as B6) mice greater than 6-weeks old were used. To generate Setdb1cKO (Setdb1flox/flox: Ddx4-CreTg/+) mice, a male carrying the Setdb1flox/+: Ddx4-CreTg/+ alleles was crossed with a female carrying the Setdb1flox/flox allele. Males carrying the Setdb1flox/+; Ddx4-CreTg/+ alleles obtained from the same letter were used as littermate controls.  The control and Setdb1cKO mice greater than 3 weeks of age were harvested and analyzed. |
| Wild animals | No wild animals were used in this study. |
| Field-collected samples | No field collected samples were used in this study. |
| Ethics oversight | All experimental work was approved by the Institutional Animal Care and Use Committee under protocol No. 21931 at UC Davis. |

Note that full information on the approval of the study protocol must also be provided in the manuscript.
